# Motor and functional outcome of selective dorsal rhizotomy in children with spastic diplegia at 12 and 24 months of follow-up

**DOI:** 10.1007/s00701-021-04954-5

**Published:** 2021-08-21

**Authors:** Tarik Alp Sargut, Hannes Haberl, Simone Wolter, Sascha Tafelski, Anne van Riesen, Maijana Linhard, Angela M. Kaindl, Ulrich-Wilhelm Thomale, Matthias Schulz

**Affiliations:** 1 Pediatric Neurosurgery, Charité - Universitätsmedizin Berlin, Berlin, Germany; 2 Division of Pediatric Neurosurgery, Universitätsklinikum Bonn, Bonn, Germany; 3 Department of Anesthesiology and Operative Intensive Care Medicine, Charité - Universitätsmedizin Berlin, Berlin, Germany; 4 Center for Chronically Sick Children, Charité - Universitätsmedizin Berlin, Berlin, Germany; 5 Department of Pediatric Neurology, Charité - Universitätsmedizin Berlin, Berlin, Germany; 6 Institute of Cell Biology and Neurobiology, Charité - Universitätsmedizin Berlin, Berlin, Germany

**Keywords:** Cerebral palsy, Spasticity, Selective dorsal rhizotomy, Modified Ashworth Scale

## Abstract

**Background:**

Selective dorsal rhizotomy (SDR) in ambulatory children affected by cerebral palsy (CP) is a surgical treatment option to lower spasticity and thereby improve gait and ambulation. The aim of the current study is to investigate the outcome of children with respect to spasticity, muscle strength, and overall function after SDR.

**Methods:**

All children who underwent SDR via a single-level laminotomy in the time period from January 2007 to April 2015 at our center were enrolled in this study. Within a standardized evaluation process, the following was assessed routinely pre-operatively and 12 and 24 months following surgery: extent of spasticity at hip adductors and hamstrings as characterized by the Modified Ashworth Scale (MAS), maximal muscle strength as characterized by the Medical Council Research Scale (MRC), overall function regarding ambulation as characterized by the Gross Motors Function Classification System (GFMCS), and overall function as characterized by the Gross Motor Function Measure (GMFM-88).

**Results:**

Matching sets of pre- and post-operative assessments of the chosen outcome parameters were available for 109 of the 150 children who underwent SDR within the observation period. After 24 months, the MAS scores of hip adductors (*n* = 59) improved in 71% and 76% of children on the right and left side, respectively. In 20% and 19%, it remained unchanged and worsened in 9% and 5% of children on the right and left side, respectively (*p* < 0.00625). For hamstrings, the rates for the right and left sides were 81% and 79% improvement, 16% and 16% unchanged, and 4% and 5% worsened, respectively (*p* < 0.00625). Muscle strength of ankle dorsiflexion and knee extension significantly improved after 24 months. Overall function assessed by GMFM-88 improved significantly by 4% after 12 months (*n* = 77) and by 7% after 24 months (*n* = 56, *p* < 0.0001).

**Conclusions:**

The presented data underlines the benefit of SDR in a pediatric patient collective with bilateral spastic CP. The procedure resulted in an effective and permanent reduction of spasticity and improved overall function without causing relevant weakness of the lower extremities.

## Introduction

Cerebral palsy (CP) is one of the most common neurological disorders in childhood worldwide with an incidence of 2–4 per 1000 live births [2]. The underlying pathophysiological mechanism is a damage during early brain development in the prenatal or perinatal period of life. According to the predominant movement disorder, spastic, dystonic, atactic, or mixed clinical presentation are distinguished [4]. The spastic subtype is the most common and can lead to an impaired mobility or inability to ambulate [28]. The ability to ambulate is commonly graded according to the Gross Motor Function Classification System (GMFCS, [20]) and detailed scales such as the Gross Motor Function Measure (GMFM-88, [30]) document functional ability and changes over a period of time or changes due to an intervention.

Alleviation of spasticity and impaired ambulation as well as improvement of the resulting delayed motor development require a multidisciplinary and multimodal approach involving physiotherapy, occupational therapy, oral medication or intrathecal application of baclofen, and intramuscular botulinum toxin injections. The introduction of selective dorsal rhizotomy (SDR) widened the armamentarium of treatment options. This procedure constitutes of a surgical intervention to transect a portion of each lumbar sensory nerve root bilaterally to reduce lower extremities’ spasticity and originates from the pioneering work of Sherrington and Foerster among others [11, 31]. The surgical procedure of SDR [37] involves either a multisegmental laminotomy [25, 26] or a tailored interlaminar access through two or three interlaminar spaces [13, 32] to address the sensory nerve roots at their neuroforaminal exit. Alternatively, a monosegmental laminotomy overlying the conus to perform the transection in proximity of the dorsal root entry was introduced by Park et al. [21, 23]. Regardless of the surgical approach, depending on the institutional protocol between 20 and 70% of the volume of each sensory nerve root from L1 to S1 (or S2) are transected bilaterally. The aim of the current study is to investigate the outcome of children with respect to spasticity, muscle strength, and overall function 12 and 24 months after SDR.

## Material and methods

### Patients and study design

For this retrospective cohort study, 150 children who underwent a SDR procedure in the time period from 01/2007 to 04/2015 were enrolled. The study was approved by the local ethics committee (EA1/138/11, EA2/167/16). Patient selection was based on an assessment by a multidisciplinary team of pediatric neurologists, pediatric neurosurgeons, pediatric orthopedic surgeons, and physiotherapists. The criteria defined by Peacock and Staudt were used to select children to undergo SDR [27]. Accordingly, candidates suitable for the procedure presented with spastic, bilateral paraparesis of the lower extremities and predominately represented grade I, II, or III of the Gross Motor Function Classification System (GMFCS). Motivation to ambulate and sufficient cognitive skills to actively participate in the mandatory post-operative rehabilitation constituted further prerequisites. Exclusion criteria were inability to ambulate (GMFCS grade V) presence of severe contractures or joint dislocation, function-limiting deformities or contractures of the skeletal system, and previous operations on the spinal column over several segments.

The surgical procedure was performed via a single level laminotomy according to the technique described by Park et al. [21, 22]. A pre-operative MRI of the spine was used to identify the position of the conus and the respective overlying lamina intended for laminotomy (usually the L1 lamina, less frequent L2 or Th12). Intraoperatively, the access via the performed laminotomy exposed the distal conus and the lowest exiting (sacral) nerve roots. The sacral nerve roots lower than S1 were covered by a microsurgical patty inserted between the estimated S1 and S2 sensory rootlets (later verified by intraoperative EMG). The performed single-level L1 (or L2) laminotomy furthermore allowed for exact identification of the L1 sensory root a either the caudal (or cranial) exposed neuroforamen, thereby defining all exposed sensory nerve roots in-between itself and the caudally inserted patty as the L2 to S1 roots — positioned in an orientation from lateral to medial. After transection of 50% of the sensory L1 root, the 5 separate sensory nerve roots L2 to S1 were then partially transected following their anatomical position from lateral to medial. The correct assignment of the treated nerve root to the assumed topographical level was verified using stimulated EMG and evaluation of the respective motor responses. For the first children of the series, this was further confirmed by simultaneous direct clinical assessment of the motor response. Uniformly, 50 to 70% of the volume of all lumbar (L1 to L5) and of S1 sensory nerve root were transected bilaterally. Starting in 2011 with the 84th patient of this series, approximately 50% of the sensory S2 root volume was bilaterally transected as well. The correct differentiation of S1 and S2 — and possible adjustment of the above-mentioned anatomical identification — was based on stimulated EMG with characteristic S2 response of abductor hallucis muscle and on the distribution of responses of the external anal sphincter between the two nerve roots [33]. Pudendal dorsal-root action potential (pDRAP) was recorded after respective peripheral stimulation from S1, S2, and S3 by placement of two recording electrodes in close proximity the nerve exits at the conus [8, 16]. While never a significant amount of pPDAP was measured for S1, its recording allowed the preservation of bladder innervation either in the non-transected part of S2 or in S3. Post-operative care included appropriate analgesia and mobilization after post-operative day 2. Children were transferred routinely to a pediatric rehabilitation facility for an extensive inpatient rehabilitation program 1 week after surgery. Follow-up examinations were performed 6, 12, and 24 months post-operatively.

### Outcome measurements

Pre- and post-surgical assessment consisted of a medical history, clinical examination, and evaluation of the following standardized scales: (i) Muscle strength was recorded according to the Medical Research Council (MRC) Scale for Muscle strength from grade 0 to 5 [18, 24]. (ii) Spasticity was recorded using the 6-point Modified Ashworth Scale (MAS, [3, 5]). (iii) Overall motor function regarding ambulation was graded according to the 5-grade Gross Motors Function Classification System (GFMCS). (iv) The Gross Motor Function Measure (GMFM-88) was utilized as a measure to evaluate overall motor function. The GMFM-88 is a validated score which computes the performance in 88 tasks in 5 major subgroups and yields an overall performance score from 0% to a maximum of 100%.

### Statistics

MAS scores of the bilateral adductors and hamstring (ischiocrural) muscles to assess spasticity and MRC scores of bilateral dorsiflexion of the ankle and extension of the knee joint to assess muscle strength were chosen as outcome measures for statistical analysis. Values of paired samples of pre- and post-operative MAS and MRC and changes in GMFM-88 were analyzed by the Wilcoxon matched-pairs signed rank test. A level of *p* < 0.05 was considered to indicate statistical significance for changes of GMFM-88. Significance level for changes of MAS and MRC scores were adjusted with Bonferroni’s correction to indicate significance at a *p* < 0.00625. Standard statistical software was used for all calculations (SPSS Statistics 25, IBM, USA).

## Results

Within the cohort of 150 patients who had undergone SDR during the observation period, a pre- vs. post-operative comparison with available matching sets of pre- and post-operative data was possible for 109 patients, 48 girls (44%) and 61 boys (56%). The mean age at the time of surgery of these 109 patients was 6 ± 2.73 years (range 2–17 years). The majority of children (83/109, 76%) were born prematurely (Table 1). For a minority of the children (20/109, 18%), cranial MRIs were available, all confirming the presence of periventricular leucomalacia (PVL).

### Spasticity

#### Adductors

A complete pre- and 12-month post-operative examination of the adductor spasticity on both sides was performed in 85 patients. MAS values had improved 12-month post-operatively in 58 children (68%) with respect to the right-sided adductors and in 61 children (72%) for the left-sided adductors. The MAS values had worsened in 3 (4%) children for the right and in 5 (6%) children for the left side. MAS values were unchanged in 24 (28%) and 19 (22%) children for the right and left adductors, respectively.

**Table 1:** Demographic data

Observation period	01/2007 to 04/2015
SDR patients (total)	*n* = 150
SDR patients (with pre-and post-operative data, included for evaluation)	*n* = 109
Female/male	48/61
Age (mean ± SD, *n* = 109)	6 ± 2.73
Premature birth	83 of 109

**Fig. 1:**
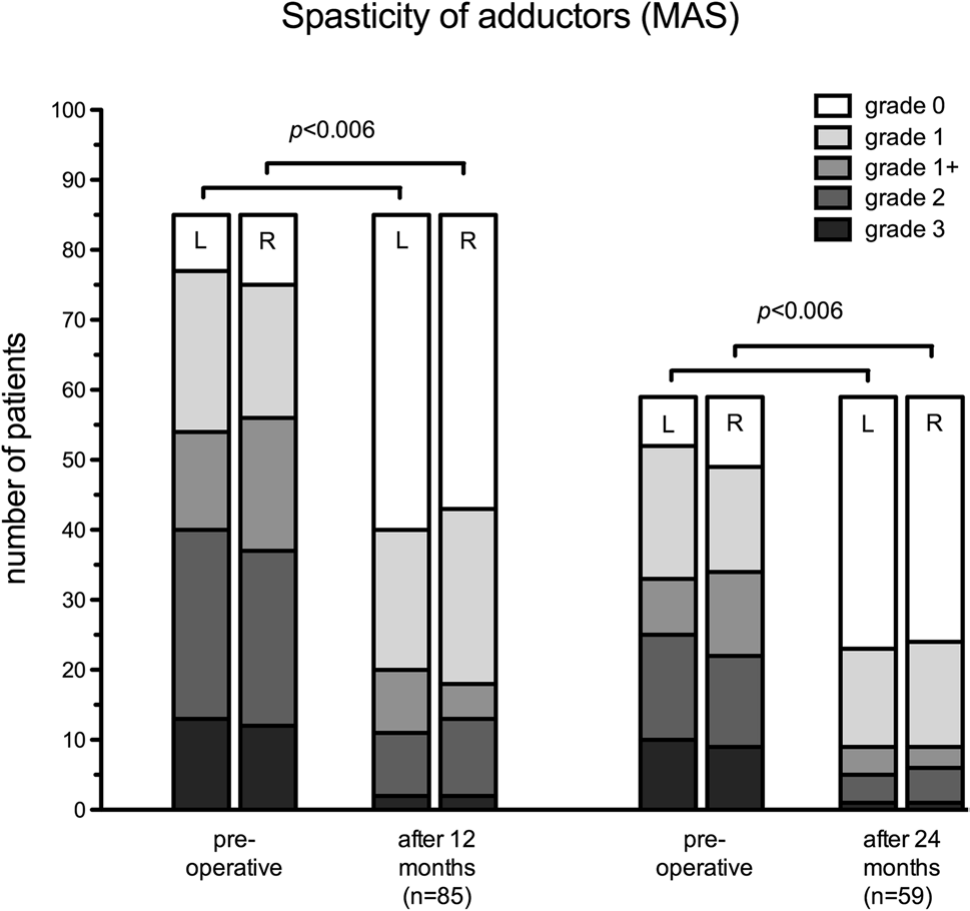
Development of Modified Ashworth Scores distribution of bilateral adductors 12 and 24 months after SDR demonstrates a significantly reduced spasticity

After 24 months, scores for 59 children were available of whom 42 (71%) and 45 (76%) demonstrated right and left side improvement, respectively. The MAS of 12 (20%) and 11 (19%) children remained unchanged on the right and left side, respectively. It worsened in 5 (9%) and 3 (5%) children on the right and left side, respectively. The proportion of children with no detectable spasticity of the adductors increased from 10 to 35 for the right and from 7 to 36 for the left side after 24 months. The overall improvement in MAS after 24 months was bilaterally significant (*p* < 0.00625, Fig. 1).

#### Hamstrings

A similar improvement was seen for the MAS of the hamstring muscles of 85 patients with available pre- and post-operative data. MAS values had improved 12-month post-operatively in 69 (81%) and 71 (83%) children with respect to the right and left hamstrings, respectively. For 15 (18%) and 11 (13%) children, the right and left MAS score remained unchanged, respectively. The MAS worsened in one child (1%) for the right and in 3 children (4%) for the left hamstrings.

**Fig. 2:**
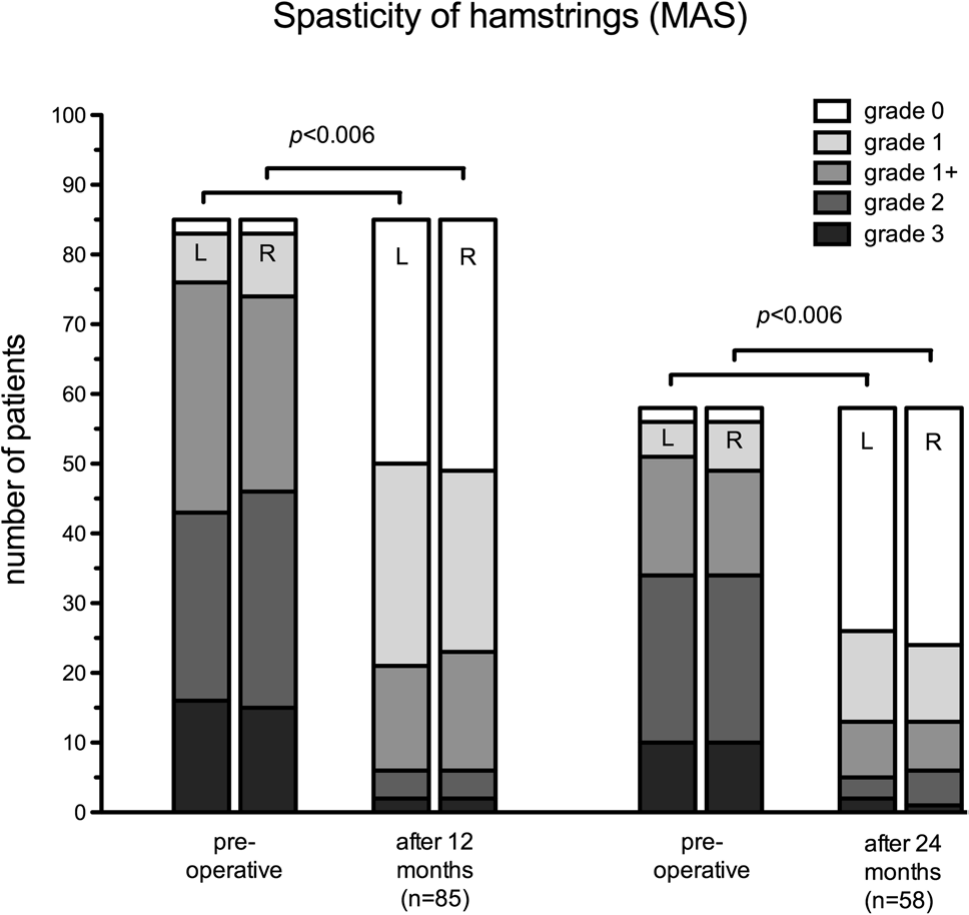
Development of Modified Ashworth Scores distribution of bilateral hamstrings 12 and 24 months after SDR demonstrates a significantly reduced spasticity

After 24 months, the MAS scores of the hamstrings were available for 58 children of whom 47 (81%) and 46 (79%) improved on the right and left side, respectively. The MAS of 9 (16%) remained unchanged on both the right and the left side, and it worsened on the left in 2 (3%) and on the right in 3 (5%) children. The proportion of children with no detectable spasticity of the hamstrings increased from 2 to 34 for the right and from 2 to 32 for the left side after 24 months. The change of MAS was significant bilaterally after 24 months (*p* < 0.00625, Fig. 2).

### Muscle strength

**Fig. 3:**
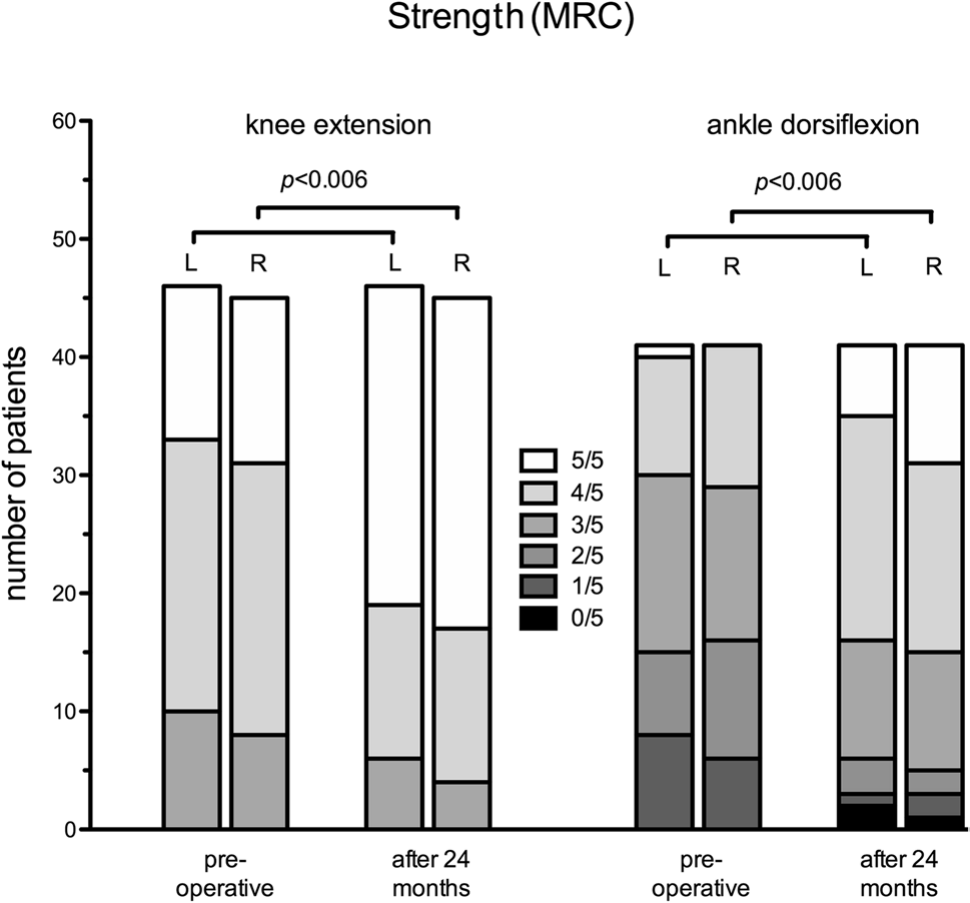
Significantly improved strength of bilateral knee extension and ankle dorsiflexion 24 months after SDR

Pre- and post-operative manual muscle strength values of the right and left ankle dorsiflexion were available for 41 children and demonstrated significantly improved strength on both sides after 24 months (*p* < 0.00625, Fig. 3). Likewise, for 46 and 45 children, comparison of pre- and post-operative strength of knee extension demonstrated a significant improvement of bilateral strength after 24 months (*p* < 0.00625, Fig. 3). Regarding the strength of further muscle groups such as hip flexion and extension, knee flexion, and ankle flexion, this improved or remained stable in a majority of children. Only in a minority of children the post-operative MRC scores were lower than the pre-operative values (Table 2).

**Table 2:** Development of muscle strength (MRC score) after 24 months

		Improved	Unchanged	Worse	*p*
Hip	Extension right (*n* = 37)	17 (46%)	11 (30%)	9 (24%)	
	Extension left (*n* = 37)	18 (49%)	15 (40%)	4 (11%)	
	Flexion right (*n* = 47)	18 (38%)	19 (40%)	10 (21%)	
	Flexion left (*n* = 47)	16 (34%)	22 (47%)	9 (19%)	
Knee	Extension right (*n* = 46)	20 (43%)	22 (48%)	4 (9%)	*p* < 0.00625
	Extension left (*n* = 45)	18 (40%)	24 (53%)	3 (7%)	*p* < 0.00625
	Flexion right (*n* = 46)	13 (28%)	24 (52%)	9 (20%)	
	Flexion left (*n* = 46)	10 (22%)	26 (57%)	10 (22%)	
Ankle	Extension right (*n* = 41)	23 (56%)	14 (34%)	4 (10%)	*p* < 0.00625
	Extension left (*n* = 41)	23 (56%)	14 (34%)	4 (10%)	*p* < 0.00626
	Flexion right (*n* = 33)	15 (46%)	7 (21%)	11 (33%)	
	Flexion left (*n* = 33)	13 (39%)	7 (21%)	13 (39%)	

### Overall functional outcomes — GMFM88 and GMFCS

**Fig. 4:**
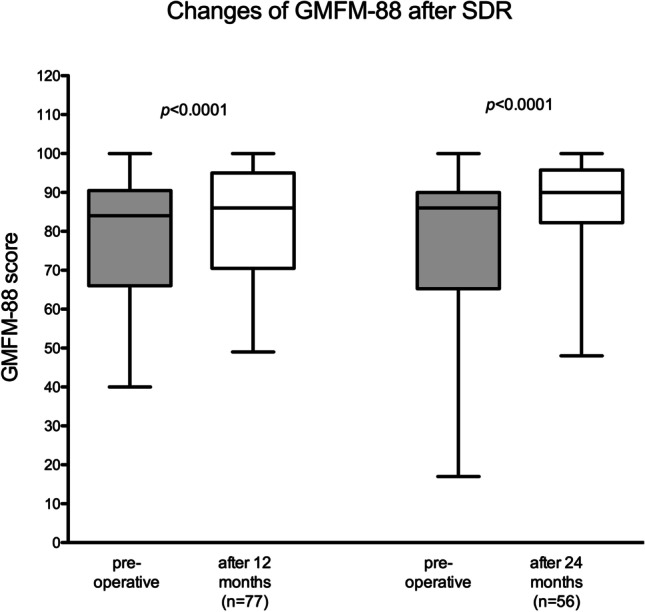
Significantly improved GMFM-Scores 12 and 24 months after SDR

**Fig. 5:**
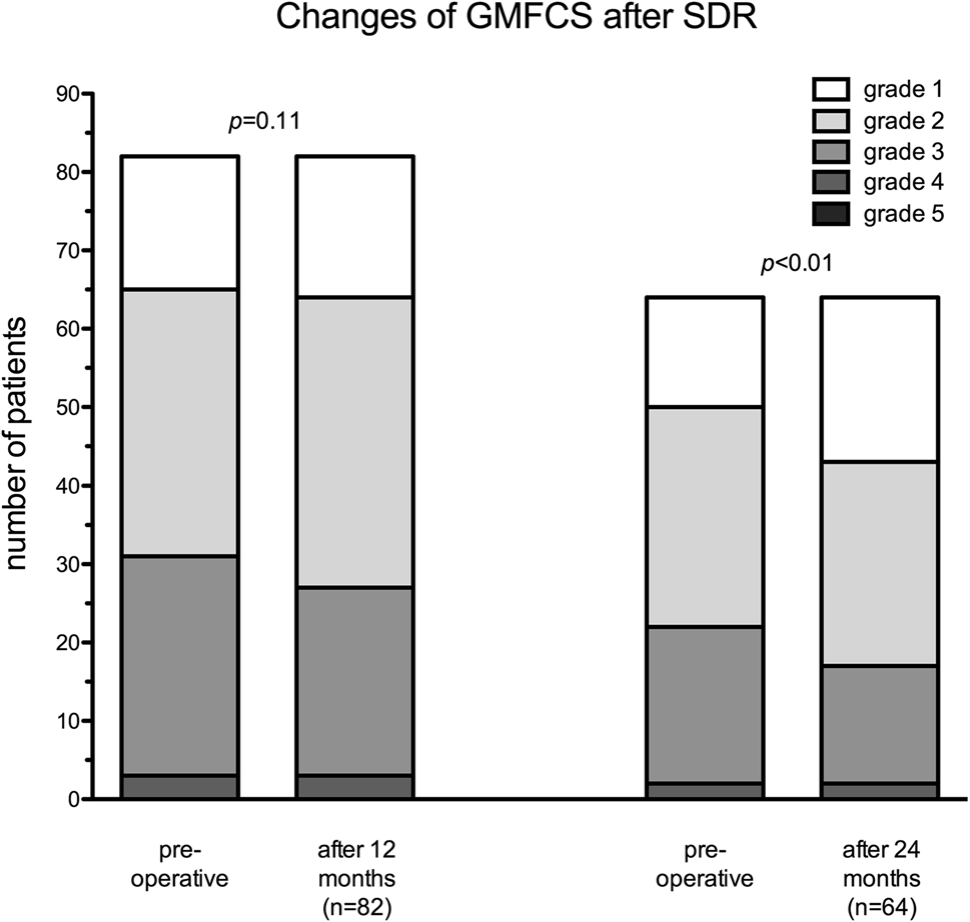
Development of GMFCS 12 and 24 months after SDR

Pre- and 12-month post-operative GMFM-88 values were available for 77 children. By 12 months after surgery, the mean GMFM-88 had increased significantly by 4% (95% confidence interval [2.0–5.4]) from an average of 79 to 83 (*p* < 0.0001). After 24 months, an even stronger increase of the GMFM-88 by 7% (95% confidence interval [5.0–9.4]) from 79 to 86 was observed (*n* = 56) (*p* < 0.0001, Fig. 4). GMFCS level data were available for 64 patients 24 months post-operatively. The majority of children (78%) remained at the same GMFCS level at follow-up 2 years post-operative, yet 20% improved by one level and one child (2%) deteriorated after 24 months (*p* < 0.01, Fig. 5). After 12 months, only 5% had already improved their GMFCS level (*p* = 0.11).

## Discussion

Here we describe the outcome of a large ambulatory pediatric patient cohort with bilateral, spastic CP following SDR via a monosegmental laminotomy. The study focuses on spasticity, functional status, and muscle strength. As main findings, we highlight 24 months after SDR reduced spasticity of bilateral adductor and hamstring muscles, improved functional score by 7% in the GMFM-88 test battery, and significantly increased strength of bilateral knee extension and ankle dorsiflexion.

### Overall function

Since the re-emergence of the SDR procedure at the middle of the last century and after many refinements, this procedure has become a relevant and unique option in the treatment of children who are affected by spastic CP. The procedure differs from other options to treat spasticity, because it targets to treat spasticity at neuronal rather than the muscular level. The transection of sensory rootlets that demonstrated exacerbated EMG-responses through their intramedullary and spinal relay during intraoperative testing alters muscle tone and reduces spasticity. The reduction of spasticity establishes the opportunity to improve gait and ambulation which were previously hindered by its presence. Therefore, SDR usually does not stand alone but is embedded in a program of intense physiotherapy and rehabilitation to utilize the established potential of less spasticity [19, 29]. The classical indication for SDR is ambulatory children — usually classified as GMFCS I–III — with a spastic bilateral presentation of CP. The intention of the procedure is to improve ambulation and gait. However, the indication to perform SDR has been widened to include more severely affected, non-ambulatory children of GMFCS IV and V as an alternative to intrathecal Baclofen [17]. For those children, the aim of the procedure would be alleviation of daily care; a progression to an ambulatory state cannot be expected. The rate of children who change their level at the GFMCS scale is usually considered to be low; commonly the children improve within their pre-operative GMFCS level. However, a portion of children of this cohort changed on the GMFCS scale to a lower (better) grade — 5% after 12 months and 20% after 24 months. Throughout literature, the rate of change of GMFCS level is variable from 4.8% after 12 months and up to 23% after longer follow-up [1, 7]. To compare the results of the presented cohort, even longer follow-up periods will be needed.

As a measurement for overall function, the GMFM-88 score was evaluated pre- and post-operatively. An increase by 4% and 7% was demonstrated after 12 and 24 months, respectively. After 20 months, an increase by 5% has been published by Engsberg et al., which is similar to the presented results [10]. Regarding the long-term development of GMFM-88, divergent results have been published. An increase of GMFM-88 over the follow-up of 1 to 5 years, followed by stable score up to15 years post-SDR, was reported by Dudley et al. [9]. On the contrary, some of the publications report the largest increase of GMFM-88 at 3–5 years post-operatively, followed by a decline to still significantly better than pre-operative scores at the long-term follow-up of more than 10 years [1, 6, 36]. Given the relatively short follow-up of 24 months of the presented patient cohort, progressive gain of function during the next years could be expected.

### Spasticity

Spasticity constitutes the clinical target symptom for SDR; its presence negatively influences ambulation, and its reduction improves overall function. The MAS (like the MRC scale) is an ordinal and not an interval scale. Therefore, the evaluation of the numeric amount of absolute changes (differences of pre- and post-operative means on those scales) — although often published, e.g., for MAS [1, 15] — is not statistically sound and is therefore omitted in the presented manuscript. Although the absolute amount of reduced spasticity cannot be calculated by the used MAS score, the overall development of spasticity is well pictured by pre- and post-operative comparison of those scores. For the two evaluated muscle groups, a majority of patients changed score within the MAS scale to a significantly better level after 24 months. This was the case for 71% and 76% for right and left hip adductors, respectively, and for 81% and 79% for right and left hamstrings, respectively. Apparently, there are data indicating that the post-operative course of spasticity of hip adductors and other muscle groups might differ. For hip adductors, several publications describe a lasting reduction of spasticity even after more than 10 years after SDR. For knee extension and ankle flexion, lower MAS scores in the early years after SDR than after long-term follow-up have been reported, which would indicate partial recurrence of spasticity. This would be in accordance with the slightly lower GMFM scores and could explain this observation. The explanation for the apparent worsening of spasticity remains yet unexplained, but could be related to puberty-induced changes of physical appearance. The 24-month follow-up of the presented patient cohort does not allow statements about long-term development of spasticity for this cohort; the evaluation of future data needs to be awaited.

### Muscle strength

One major concern towards SDR is the apprehension to elicit muscular weakness after the procedure. The decreased tone in the anti-gravity musculature of the lower extremities can produce an impression of muscular weakness and impaired security when ambulating immediately post-operatively. This represents rather the unmasked true underlying muscular strength together with a possibly altered proprioception than elicited weakness by the SDR procedure. Clinically, this situation is usually limited to a few weeks after which this impression subsides. Correspondingly, the data of the presented cohort demonstrated a significant improvement of the primary endpoint muscles for knee extension and ankle dorsiflexion after 24 months. For all other examined muscle groups, a majority of patients either improved or remained stable, only a minority demonstrated lower MRC values at the 24-month post-operative examination. This appears to be in line with previous publication which has documented improved strength in several lower extremities’ muscle groups [1, 10, 15]. Therefore, the presented data of the cohort along with the literature rather demonstrate an improved muscular strength after SDR and disprove the assumption of impaired strength as an argument against this surgical procedure.

### Operative technique

The operative access to the sensory lumbar and sacral nerve roots was achieved via a one level laminoplasty centered over the conus position as compared to a 5-level laminectomy or laminoplasty. Previously published data of this patient cohort indicated a lesser incidence of mild scoliosis of 10% as compared to a 55% rate after 5-level laminectomy [14, 34, 35]. The 10% incidence would be comparable with the natural incidence of scoliosis of ambulatory children affected by CP [12]. Since the reduction of spasticity and improvement of overall function seem to be independent from surgical access, the single-level laminotomy should be preferred because of the better risk profile regarding secondary spinal deformity. Alternatively, the described keyhole access for a SDR through three interlaminar spaces will likewise carry significantly lower risk for developing a post-operative spinal deformity compared to multilevel laminotomies [13, 32].

### Limitations

The lack of a control group does not allow for direct comparison and allows only a descriptive statistical evaluation of the patient cohort. However, given the multiplicity of reports about efficiency of SDR, the set-up of a randomized controlled trial would be problematic, because it would deprive a group of children off this treatment option and parents’ consent to such a study design would be questionable. For 109 of 150 children, who underwent SDR during the examination period, matched sets of pre- and post-operative data of the evaluated data (MAS, MRC, GMFCS, and GMFM-88 scores) were available. There are several contributing and explaining factors regarding missing data. The over-regional recruitment for the SDR procedure resulted in the loss of some follow-up data for evaluation for those children who opted to be followed up at their referring pediatric institution. A further limiting factor is the need for cooperation of children during evaluation and examination. The investigation of all items, especially given the multiplicity of tasks for the GMFM-88, is a time-consuming process and requires full concentration of the child for more than 1 h. Due to the underlying condition of the participating children with associated neurocognitive impairment, full cooperation was not always possible, which resulted in a relevant amount of incomplete data sets despite high compliance and attendance of all follow-up appointments.

Furthermore, the objective of SDR is to reduce spasticity and to positively impact on overall function in the long term. Obviously, since the clinical follow-up of the presented patient cohort is currently only 24 months, no statement about the long-term development of this cohort can be made and further follow-up needs to be awaited.

## Conclusion

The presented data confirm that the selected patient collective with the diagnosis of bilateral, spastic CP in a single-center protocol benefitted from SDR after 24 months. The procedure resulted in an effective and permanent reduction of spasticity and improved overall function without causing relevant weakness during the evaluated follow-up.
